# Synergy Between Dietary Quercetin and Vitamin E Supplementation in Aged Hen’s Diet Improves Hatching Traits, Embryo Quality, and Antioxidant Capacity of Chicks Hatched From Eggs Subjected to Prolonged Storage

**DOI:** 10.3389/fphys.2022.873551

**Published:** 2022-04-11

**Authors:** Felix Kwame Amevor, Zhifu Cui, Xiaxia Du, Zifan Ning, Xun Deng, Dan Xu, Youhao Wu, Xueqing Cao, Shuo Wei, Gang Shu, Xue Han, Yaofu Tian, Diyan Li, Yan Wang, Yao Zhang, Xiaohui Du, Qing Zhu, Xiaoling Zhao

**Affiliations:** ^1^ Farm Animal Genetic Resources Exploration and Innovation Key Laboratory of Sichuan Province, Sichuan Agricultural University, Chengdu, China; ^2^ Department of Basic Veterinary Medicine, Sichuan Agricultural University, Chengdu, China; ^3^ Institute of Animal Husbandry and Veterinary Medicine, Guizhou Academy of Agricultural Sciences, Guiyang, China

**Keywords:** egg storage, offspring, dietary quercetin, vitamin E, antioxidant capacity

## Abstract

The current study aims to investigate the effects of the synergy between quercetin and vitamin E in aged hen’s diet on hatchability and antioxidant levels of the embryo and newly hatched chicks from prolonged storage eggs. A total of 400 breeder laying hens of 65 weeks of age were selected and randomly divided into 4 groups. Birds were fed a basal diet alone (Control), and basal diets supplemented with quercetin (Q) (0.4 g/kg) and vitamin E (VE) (0.2 g/kg) alone and their combination (0.4 g/kg Q + 0.2 g/kg VE) for 14 weeks, respectively, to determine their effects on yolk antioxidant status, fertility, embryonic mortality, hatchability, antioxidant status of embryonic tissues, as well as the antioxidant status of the newly hatched chicks. The results showed that the hen’s dietary Q + VE increased the yolk weight, as well as increased the antioxidant status of the egg yolk (*p* < 0.05). Compared with the control group, the supplementation of Q + VE significantly increased the hatchability of set-fertile eggs and decreased early embryonic mortality in eggs stored for 7 and 14 days, respectively (*p* < 0.05), and also improved the antioxidant capacity of the embryos obtained from eggs stored for 14 days (before incubation) (*p* < 0.05). Moreover, Q + VE increased the levels of SOD, GSH-Px, T-AOC, T-SOD, and CAT in the liver, heart, and pectoral muscle of the embryo, 1-day-old and 14-day-old chicks (*p* < 0.05), as well as upregulated the antioxidant related genes (GPx-1, GPx-2, GPx-4, DIO-1, and SOD-1) in the liver of the embryo, 1-day-old and 14-day-old chicks hatched from 14-days storage eggs (*p* < 0.05). Meanwhile, the MDA levels were decreased by the Q + VE in the embryo and post-hatched chicks (*p* < 0.05). In conclusion, these findings suggested that maternal dietary Q + VE exerts beneficial synergistic effects on the antioxidant capacity of the egg yolk, embryo, and chicks during prolong egg storage, therefore, Q + VE could be used as a dietary measure to enhance hatchability and chick quality in poultry production.

## Introduction

Fertility is an important parameter in birds, that reflects the actual reproductive capacity of breeder hens. Various factors such as egg storage period, age, breed, pre-incubation, lighting, level of nutrition, mating, and time of mating, influence fertility in birds (Miazi et al., 2012). Moreover, hatchability is a trait of economic importance in the chicken industry because of its strong effect on chick output (Wolc et al., 2010). Good hatchability of eggs to some extent is heritable, which is determined by genotype and environment (Peters et al., 2008).

In chicken production, nutrients are more effectively transferred from the mother to the embryo and offspring, which indicates efficient maternal nutrition (Wang et al., 2021). However, in breeder hens, age negatively affects feed conversion ratio (feed-egg ratio), due to excessive oxidative stress, which obviously affects reproductive organ function and quality egg production (Amevor et al., 2021a, b). Moreover, in aged animals, the physiological functions of the gastrointestinal tract (GIT) are reduced and prone to invasion by pathogens and inflammation, hence, cause a reduction in nutrient utilization. This is because most aged animals have disrupted intestinal morphology (Lin et al., 2017; Takiishi et al., 2017; Mishra and Jha, 2019). Therefore, maternal age and nutrition have an important influence on the embryonic development and growth of offspring in poultry production (Miazi et al., 2012), thus, hen’s nutrition significantly influenced chick quality (Emamverdi et al., 2019). Owing to this, the diet for breeder hens should have high quality and sufficient amount of nutrients to maintain optimal reproductive performance and improve chick quality.

Generally, in poultry, there are minimal adverse effects on hatchability if eggs are stored less than 7 days (Saito and Kita, 2011; Gharib, 2013). However, a greater prolongation of egg storage duration beyond 7-days results in decreasing hatchability, partially due to increased early embryonic mortality (Dymond et al., 2013; Gharib, 2013), and also decreased the quality and performance of chick’s post-hatch (Hamidu et al., 2011; Gharib, 2013). Since, the quality of eggs from aged hens is decreased (Amevor et al., 2021b), it is obvious that the viability of such eggs could be negatively affected when subjected to prolong storage. Previous study has shown that shell color and light or darkness affects hatchability and chick quality, for instance, hatchability of spotted brown eggs incubated under darkness or lighting (Farghly et al., 2015). For now, it is not certain whether the negative effects of prolong egg storage such as increased embryonic mortality and decreased hatchability are caused by the effects of high production and accumulation (oxidative stress) of reactive oxygen species (ROS) and lipid peroxidation of the blastodermal cells in the early embryo. Therefore, it is important for animal nutritionist to develop dietary supplements (antioxidants) that can protect the egg yolk, thereby attenuating embryonic mortality to improve hatchability.

The antioxidant activity of flavonoids is attributed to one or more aromatic hydroxyl groups contained in their molecule, which actively scavenge free radicals (Robbins, 2003; Shu et al., 2020). Quercetin (Q) is a phytochemical compound that belongs to the subclass of flavonols and is commonly found in many fruits and vegetables (Peschel et al., 2006; Liu et al., 2013; Amevor et al., 2021a, b). In other studies, quercetin showed an anti-aging property, due to its cytoprotective actions that promotes early embryonic development of oocytes in mice and humans (Cao et al., 2020). Dietary quercetin improves meat oxidative stability in broiler chickens (Goliomytis et al., 2014); and also possesses immunomodulatory property, by enhancing the production of IgY and IgA antibodies in birds (Hager-Theodorides et al., 2014; Ognik et al., 2016). Utilization of quercetin by laying hens improves their antioxidant status (Iskender et al., 2016), reduce yolk (Liu et al., 2013) and serum cholesterol levels (Iskender et al., 2016).

Vitamin E (VE) is a strong lipid-soluble antioxidant that plays important role in protecting cell membranes through breaking peroxide chains, and preventing oxidation of polyunsaturated fatty acids (Siegel et al., 2001; Surai et al., 2016). A study demonstrated that during summer periods, the supplementation of dietary vitamin E, A, and selenium, and their combination promote feed intake and feed conversion in laying hens (Mahrose et al., 2012). Previous studies have showed that VE exerts health benefits on breeder hens (Zaghari et al., 2013; Urso et al., 2015), and also attenuates lipid peroxidation in both egg yolk and embryo (Tsai et al., 2008; Jiang et al., 2013). In addition, the synergy of antioxidants such as vitamin E and selenium promotes production performance in laying hens by alleviating the impacts of heat stress (Abd El-Hack et al., 2017).

To the best of our knowledge, no work has been yet conducted to elucidate the effects of the synergy between dietary quercetin and vitamin E supplementation in hen’s (maternal) diet on the fertility, hatchability, and antioxidant capacity of embryo and chicks hatched from eggs subjected to prolonged storage. However, it remains unclear whether or not the combination of hen’s (maternal) dietary Q + VE supplementation could synergistically increase the breeder egg resistance to oxidative stress caused by prolonged storage. Therefore, this study, aimed to evaluate the synergistic effects of hen’s dietary Q + VE on fertility, embryo quality, hatchability, chick quality, and antioxidant capacity of chicks hatched from eggs subjected to prolonged storage times.

## Materials and Methods

### Experimental Design and Diets

A total of 400 Tianfu breeder laying hens of 65 weeks of age (a meat-type chicken, obtained from the Poultry Breeding Farm of Sichuan Agricultural University, China) were randomly selected and allotted into 4 treatment groups (100 hens each) consisting of 4 replicates of 25 chickens each. “Tianfu breeder chicken” is a fast and high-quality jute feathered green foot type quality chicken developed by the Poultry Research Breeding Group of Sichuan Agricultural University and Sichuan Banghe Agricultural Science and Technology Co., Ltd. Sichuan, China. The Tianfu breeder chickens (meat-type and egg-type) are highly productive local indigenous chicken breed characterized with fast growth rates. The productive age of Tianfu breeder hens is approximately 180–300 days, however, their productivity declines gradually and falls by less than 60% after 60 weeks (Tian et al., 2021).

The aged breeder hens were fed a basal diet alone (Control group), basal diet supplemented with quercetin (0.4 g/kg) (Q group), basal diet supplemented with vitamin E (0.2 g/kg) (VE group), and basal diet supplemented with the combination of quercetin and vitamin E (0.4 g/kg Q + 0.2 g/kg VE) (Q + VE group) to determine their effects on the yolk antioxidant status, fertility, embryonic mortality, hatchability, antioxidant status of the embryonic tissues, as well as the antioxidant status of the newly hatched chicks. Quercetin and Vitamin E were supplied by Shanxi Huike Plant Development Co., Ltd (Xian, Shaanxi, China). We determined the purity (95%) of the Quercetin using High-Performance Liquid Chromatography. The experimental hens were kept in a closed type housing unit and in an individual wire cages (one hen per cage) with the following dimensions: width: 48.8 cm, depth: 38.1 cm, and height: 38.1 cm, under controlled lighting of 16L: 8D lighting, temperature of 22 ± 1°C and optimal ventilation during the 14-weeks experimental period. Throughout the 14-weeks experimental period, the hens were given 120 g feed/day/hen and water was provided *ad libitum*. The recommended levels of vitamin E and quercetin inclusion were chosen based on previous studies (Liu et al., 2013; Yang J. X. et al., 2018; Yang et al., 2020). The recommended level of vitamin E for Tianfu Broiler Breeder Hens is 30 mg/kg Chinese Standard (Chinese chicken standard, 2004). The composition and nutritional values of the basal diet fed the aged hens have been reported (Amevor et al., 2021a). The basal diet was prepared by mixing all the dietary ingredients together in a crumble form. The breeder hens were artificially inseminated every 4 days.

### Egg Yolk Characteristics and Yolk Antioxidant Status

At 3 days to the end of the experiment, 2 fresh eggs per replicate (8 eggs per treatment) were randomly selected and weighed for the determination of egg yolk characteristics. The yolk ratio was calculated using the formula: yolk weight/egg weight. Thereafter, the yolks from each replicate were mixed and stored at −20°C for subsequent analysis of antioxidant indices. Briefly, the yolk samples (0.4 g) were homogenized in 3.6 ml of ethanol for the determination of malondialdehyde (MDA) and in 1.6 ml of physiological saline for the determination of glutathione peroxidase (GSH-Px), total antioxidant capacity (T-AOC), catalase (CAT), and total superoxide dismutase (T-SOD). Thereafter, the homogenates were centrifuged (at 3,000 rpm for approximately 10 min at 4°C) to obtain the supernatant. Furthermore, the protein concentration in the supernatants was determined using a Total Protein Assay kit (Nanjing Jiancheng Bioengineering Institute, Nanjing, China). Then, the GSH-Px concentration, T-SOD activity, T-AOC, CAT, and MDA contents were determined using commercial biochemistry assay kits following the instructions provided by the manufacturer (Bioengineering Institute of Nanjing Jiancheng, China).

### Egg Collection, Storage, and Preparation for Incubation

Within 2 days to the end of the feeding trial (14th week), eggs were collected in two batches; thus, in the first batch: 20 seed eggs (sound eggs, without cracks and discoloration) were randomly collected from each replicate, totally 80 per treatment. After the collection, the eggs were stored for 7 days before incubation. Similarly, the second batch of eggs were also collected randomly just as the first batch (same quantity, 20 eggs per replicate), but the eggs (second batch) were stored for a period of 14 days before incubation. In the egg collection process, each egg was re-grouped according to the various dietary groups and replicates they belong, by proper tagging for easy identification. Moreover, within the storage, the eggs were subjected to consecutive turning at suitable periods. The storage temperature and relative humidity were controlled at 16–18°C and 50–60%, respectively. Thereafter, they were incubated in an automatic incubator at 37.5°C and 55% RH in trays identified per replicate. Proper cleaning, disinfection, and fumigation were carried out before setting of eggs.

### Setting of Hatching Eggs, and Determination of Fertility, Embryonic Mortality and Hatchability

Candling was conducted on day 10 and 18 for all the eggs under incubation to determine fertility and embryonic mortality. The process of candling was undertaken in a dark room using a Candler. The fertile eggs were differentiated by a densely clouded and opaque network of veins, indicating development of an embryo within the egg, while the infertile eggs were translucent under the light. Data on infertile eggs and embryonic mortality were recorded. On day 18 of the incubation period, 8 normal eggs were randomly selected from each treatment (2 eggs per replicate) and the embryos were removed to isolate the pectoral muscle, heart, and liver. The samples were quickly stored on ice, and subsequently stored at −20°C for further biochemical analysis. Moreover, parts of the liver samples were quickly frozen in liquid nitrogen and stored at −80°C for further RNA extraction and subsequent qRT-PCR analysis. After the candling process, the fertile eggs were transferred into the hatching tray, according to their replicates and then moved into the hatching chamber at 36.5°C and 65% RH to complete the incubation process. After the hatch, the chicks were left in the hatchery until they were 90% dried. Then on the 21st day or 21.5 days, hatchlings (including the normal, weak, and abnormal) were taken out of the hatcher, counted, and weighed using a digital weighing scale (g). The unhatched eggs were recorded. The normal and healthy chicks, having body weight of 32 g or above, were categorized as first-grade chicks. Thereafter, 8 chicks (1-day-old) were randomly selected from each treatment (2 chicks per replicate) per batch for blood sample collection, and then were euthanized and the pectoral muscle, heart, and liver tissue samples were collected. The tissue samples were stored at −20°C for biochemical analysis, whereas part of the liver sample was stored at −80°C for subsequent RNA extraction.

Furthermore, 32 chicks per treatment (8 chicks per replicate) per batch (chicks hatched from eggs stored for 7 days and those stored for 14 days) were randomly selected and raised under controlled environment for 2 weeks and were fed normal starter diet, respectively. The composition and nutrient level of the basal diet fed the chicks are presented in Table 1. Thereafter, 8 chicks per treatment per batch were randomly selected, blood sampled, and euthanized for sample (pectoral muscle, heart, and liver) collection. The samples of pectoral muscle, heart, and liver were stored at −20°C for biochemical indices analysis, and parts of the liver sample was stored at −80°C for subsequent RNA isolation and qRT-PCR analysis.

**TABLE 1:** Ingredient composition and calculated nutrient content of the basal diet (Offspring’s diet) (%, as fed-basis).

Ingredient	Content (%)	Nutrient	Content
Corn	50.86	Metabolizable energy (kcal/kg)	2,925.00
Soybean meal, 43%	30.42	Available phosphorus	0.45
Wheat flour	4.00	Crude protein	21.80
Soybean oil	2.36	Digestible methionine	0.57
Rapeseed meal	2.00	Calcium	0.95
Gluten meal	3.00	Digestible threonine	0.81
Calcium hydrophosphate	1.76	Digestible lysine	1.25
Vitamin and mineral premix^a^	0.20	Vitamin E (mg/kg)	100.00
Limestone	1.10	Digestible methionine + cystine	0.89
Corn distiller dried grains with solubles	3.00		
Threonine, 98.5%	0.14				
Sodium chloride	0.32				
Choline chloride, 50%	0.12				
DL-Methionine, 99%	0.27				
L-Lysine hydrochloride, 98.5%	0.45				
Total	100				

a Supplied per kg feed: **Chicks:** VA, 10,000 IU; VD_3_, 4000 IU; VE, 100 mg; VK_3_, 3 mg; VB12, 0.02 mg; thiamin, 3.0 mg; pyridoxine, 4 mg; riboflavin, 10 mg; niacin, 65 mg; folic acid, 2 mg; biotin, 0.2 mg; pantothenic acid, 15 mg; copper, 10 mg; iron, 80 mg; manganese, 120 mg; selenium, 0.3 mg; zinc, 100 mg; iodine, 1.0 mg.

### Antioxidant Status of Embryo and 1-day and 14-day Old Chicks

The tissues (pectoral muscle, heart, and liver) of the embryo, 1-day-old and 14-day-old chicks (stored in −20°C) were grounded and homogenized in physiological saline at a ratio of 1:9 (sample: saline), respectively, and then were centrifuged (1,500 × g, 4°C and 10 min) to obtain the supernatant for the determination of MDA, GSH-Px, T-SOD, T-AOC, and CAT using commercial biochemistry kits, following the instructions provided by the manufacturer (Bioengineering Institute of Nanjing Jiancheng, China). Moreover, MDA, GSH-Px, T-SOD, T-AOC, and CAT were also determined in the serum obtained from the 1-day and 14-day-old chicks using commercial biochemistry kits, following the manufacturer’s instructions (Bioengineering Institute of Nanjing Jiancheng, China).

### Gene Expression Abundance of GPX-1, GPX-4, DIO-1, and SOD1 in the Liver

Total RNA was extracted from the liver tissues of the embryo, day-1 and day-14-old chicks following previously described procedures (Kang et al., 2017; Cui et al., 2020; Madkour et al., 2021a; Madkour et al., 2021b) using TRIzol reagent (Takara, Dalian, China), following the instructions provided by the manufacturer. Thereafter, we determined the concentration and purity of the RNA extracted using Nanodrop 2000C (Thermo Fisher Scientific, Waltham, MA, United States) according to the absorbance ratio of A260/280. Thereafter, the singlestrand cDNA was synthesized using the PrimeScript RT Reagent Kit (Takara, Dalian, China) following the manufacturer’s guide. Then, the single-strand cDNA was used for qRT-PCR analysis using the CFX96 Real-Time System (Bio-Rad, Hercules, CA, United States) under conditions such as: 95°C for 3 min, 40 cycles of 95°C for 10 s and annealing temperature for 20 s, followed by a final extension at 72°C for 20 s, and then the melt curve analysis was performed at 65–95°C. The amplification efficiencies of the target genes ranged from 95 to 105%. Each qRT-PCR reaction was performed with the volumes of 15 µl containing 6.25 μl TB Green TM Premix (Takara), 0.3 µl forward and reverse primers, 1.5 µl cDNA, and 6.65 µl DNase/RNase-Free Deionized Water (Tiangen, Beijing, China), as previously described (Kang et al., 2017; Cui et al., 2020; Madkour et al., 2021a, b). *GAPDH* was used to normalize (endogenous control) the expression of genes, whereas the fold change in gene expression was quantified using the 2^−ΔΔCt^ method (Livak and Schmittgen, 2001), where; ΔCt = Ct target gene - Ct housekeeping gene, and ΔΔCt = ΔCt - ΔCt reference. Primer 5 software was used in designing gene-specific primers used for qRT-PCR analysis according to the coding sequences of the target genes (Table 2). All samples were measured in triplicate and the experiment was performed twice.

**TABLE 2:** Primers used for quantitative real-time polymerase chain reaction (qRT-PCR).

Gene	Sequence (5′-3′)	Product length (bp)	Annealing Temperature (°C)	Accession number
*GPX-1*	F: CTG​CAA​CCA​ATT​CGG​GCA​C R: CAC​CTC​GCA​CTT​CTC​GAA​CA	121	60.08	NM_001277853.3
*GPX-4*	F: AGAATGGCGGACGAGTGG R: CTG​TAC​TGC​TCC​AGG​GAC​AC	92	59.42	NM_001346448.2
*DIO-1*	F: CAC​GCA​GTA​GAT​GGA​TGG​G R: GCG​CTG​CGT​ATT​TTG​AGC​TG	163	60.88	NM_001097614.2
*SOD-1*	F: TGA​CCT​CGG​CAA​TGT​GAC​TG R: CAT​GGT​ACG​GCC​AAT​GAT​GC	103	60.32	NM_205064.2
*GPx-2*	F: CGC​CAA​GTC​CTT​CTA​CGA​CC R: GGT​GTA​ATC​CCT​CAC​CGT​GG	133	60.46	NM_001277854.3
*GAPDH*	F: TCCTCCACCTTTGATGCG R: GTGCCTGGCTCACTCCTT	144	60	NM_204305.1

### Statistical Analysis

All data were analyzed by one-way analysis of variance (ANOVA) using SPSS 20 Statistical Analysis Software (SPSS Inc, Chicago, IL, United States). Therefore, all experimental data are indicated as the Standard Error Mean (SEM) and differences among treatments were examined using Tukey’s test. Calculated Δ Ct (corrected sample) = mean value of target gene - mean value of internal reference gene, ΔΔ Ct = Δ Ct-mean value of control group. Values were significantly different at *p* < 0.05.

## Results

### Effects of Dietary Quercetin (Q), Vitamin E, and Combination of Q and VE on Egg Characteristics and Yolk Antioxidant Status

As shown in Table 3, the supplementation of dietary quercetin and vitamin E synergistically improved the feed intake compared to the control group. Moreover, the egg and yolk weights of the Q + VE group were significantly increased as compared to the control group (*p* < 0.05). However, there were no difference in the egg weights among the dietary supplement groups, as well as between Q and Control groups. The combination of Q and VE did not affect the yolk ratio compared to all the groups (*p* > 0.05). Furthermore, we observed (Table 4) that all the treatment groups reduced the levels of MDA in the yolk compared to the control (*p* < 0.05) at both time points, but Q + VE group reduced the MDA levels better compared to the Q group at d14. The levels of the antioxidant indices GSH-Px, T-AOC, T-SOD, and CAT in the egg yolk of the treatment groups in both time points were significantly higher than that of the control group, however, at d14 the levels of GSH-Px and CAT of the Q + VE group were significantly higher compared to the Q and VE groups, whereas T-AOC levels in the Q + VE group were higher than that in the VE group (Table 4, *p* < 0.05).

**TABLE 3:** Effects of dietary Q, VE, and Q + VE on egg characteristics and yolk antioxidant status.

Traits	Treatments				
	C	Q	VE	Q + VE	SEM
Feed intake (g/day/laying hen)	93.75^b^	95.79^b^	103.37^ab^	113.9^a^	2.61
Egg weight (g)	44.4^b^	54.2^ab^	55.3^a^	60.2^a^	1.8
Yolk weight (g)	13.9^c^	16.6^b^	16.7^b^	20.9^a^	0.7
Yolk ratio (%)	31.7^a^	30.8^a^	30.3^a^	35.2^a^	1.2

^a,b,c^ Means in the same row without a common superscript letter differed statistically (*p* < 0.05). SEM: standard error mean.

**TABLE 4:** Effects of dietary Q, VE, and Q + VE on egg yolk antioxidant status.

Parameters	Day 7					Day 14				
	C	Q	VE	Q + VE	SEM	C	Q	VE	Q + VE	SEM
MDA (nmol/g prot.)	150.0^a^	108.2^b^	102.7^b^	89.3^b^	6.4	142.7^a^	109.9^b^	105.4^bc^	88.8^c^	5.4
GSH-Px (U/g prot.)	21.1^b^	28.4^b^	29.2^b^	40.0^a^	2.0	16.4^c^	30.5^b^	30.0^b^	42.2^a^	2.5
T-AOC (μmol/g prot.)	5.0^b^	6.6^a^	6.1^ab^	7.3^a^	0.3	4.8^c^	6.8^ab^	6.4^b^	7.74^a^	0.3
T-SOD (U/g prot.)	188.7^b^	220.1^ab^	212.9^ab^	258.7^a^	9.0	199.7^b^	237.6^ab^	218.4^ab^	266.1^a^	8.3
CAT (U/g prot.)	15.5^b^	15.9^b^	17.0^ab^	22.0^a^	0.9	14.2^b^	16.0^b^	17.5^b^	22.8^a^	1.0

^a,b,c^ Means in the same row within the same day without a common superscript letter differed statistically (*p* < 0.05). SEM: Standard Error Mean. Glutathione peroxidase (GSH-Px), total antioxidant capacity (T-AOC), catalase (CAT), and total superoxide dismutase (T-SOD), malondialdehyde (MDA).

### Impacts of Q, VE, and Q + VE on Fertility, Embryonic Mortality and Hatchability

As represented in Table 5, we observed that the fertility of the hens was improved in the dietary supplement groups compared to the control group at both d7 and 14, however, the Q + VE improved the fertility better at day 7 (*p* < 0.05). In addition, we observed that Q, VE, and Q + VE significantly improved the hatchability of the set and fertile eggs, and increased the first-grade chicks compared to the control group at both time points, but the Q + VE improved the hatchability of the set eggs at d14 as compared to the individual dietary Q and VE groups (*p* < 0.05). We found that the supplementation of Q, VE, and Q + VE significantly decreased the embryonic mortality at the mid and late embryonic developmental stages, as wells as improved the body weights and relative weights compared to the control group at both time points (*p* < 0.05). No difference existed among the treatment groups at both time points (*p* > 0.05).

**TABLE 5:** Impacts of Q, VE, and Q + VE on fertility, embryonic mortality and hatchability.

Traits	Day 7					Day 14									
	C	Q	VE	Q + VE	SEM	C		Q		VE		Q + VE		SEM	
Fertility, %	89.78^c^	95.02^b^	95.30^b^	97.81^a^	0.79	84.11^b^		92.59^a^		92.56^a^		96.76^a^		1.27	
Hatchability of set eggs, %	68.80^b^	73.23^ab^	72.47^ab^	78.83^a^	1.16	64.85^c^		76.10^b^		73.35^bc^		87.02^a^		2.34	
Hatchability of fertile eggs, %	71.30^b^	76.61^ab^	75.80^ab^	82.24^a^	1.48	68.14^b^		77.39^ab^		75.21^ab^		83.75^a^		1.96	
First-grade chicks, %	79.15^b^	91.71^a^	91.11^a^	97.50^a^	2.07	74.10^b^		84.21^ab^		86.11^ab^		94.22^a^		2.48	
Embryonic mortality, %																
Mid^1^	0.82^a^	0.59^b^	0.59^b^	0.46^b^	0.15		0.82^a^		0.62^ab^		0.67^b^		0.46^b^		0.04	
Late^2^	6.21^a^	3.82^b^	3.96^b^	3.00^b^	1.41		5.86^a^		4.15^b^		3.44^b^		2.82^b^		0.33	
Chick weight, g	42.28^b^	47.81^a^	49.30^a^	50.37^a^	0.74		34.71^b^		42.62^ab^		42.87^ab^		49.21^a^		1.66	
Relative chick weight, %	64.14^b^	70.65^a^	70.08^ab^	71.27^a^	3.91		61.55^b^		71.42^a^		69.92^ab^		75.47^a^		1.66	

^a,b,c^ Means in the same row within the same day without a common superscript letter differed statistically (*p* < 0.05). SEM: Standard Error Mean. Fertility (F) % = No. of fertile eggs/total No. of eggs set × 100. Hatchability (H) % = No. of hatched chicks/total No. of eggs set × 100. Fertile (HF) % = No. of hatched chicks/total No. of fertile eggs × 100. Embryonic mortality (EM) % = No. of dead embryos/total No. of hatched chicks x 100.

### Dietary effects of Q, VE, and Q + VE on the Antioxidant Status of Embryo, and day 1 and 14 old Chicks

Table 6 represents the results of the antioxidant status of the embryo. The MDA levels of the embryo’s liver, heart, and pectoral muscle in the dietary Q, VE, and Q + VE groups were reduced significantly compared to the control group at both time points (*p* < 0.05). No difference existed among the dietary supplement groups (*p* > 0.05). Moreover, the levels of the antioxidant indices GSH-Px, T-AOC, T-SOD, and CAT of the embryo’s liver, heart and pectoral muscle in the Q + VE group were significantly higher compared to the control at both d7 and d14 (*p* > 0.05). Similarly, the MDA levels in the blood, liver, heart and pectoral muscle of the day 1 and 14 chicks were significantly reduced by the maternal dietary Q + VE compared to the control group (*p* < 0.05), whereas the levels of the antioxidant indices GSH-Px, T-AOC, T-SOD, and CAT were significantly increased by the Q + VE compared to the control group at both time points (Tables 7, 8; *p* < 0.05). These results indicated that the combination of Q and VE exert better antioxidant effects on chicks compared to the supplementation of individual Q and VE in the maternal diet.

**TABLE 6:** Dietary effects of Q, VE, and Q + VE on the antioxidant status of the embryo.

Parameters	Day 7					Day 14				
	C	Q	VE	Q + VE	SEM	C	Q	VE	Q + VE	SEM
Liver											
MDA (nmol/g prot.)	1.56^a^	1.01^ab^	0.82^b^	0.58^b^	0.12	2.09^a^	1.14^ab^	1.03^b^	0.75^b^	0.17
GSH-Px (U/g prot.)	55.88^b^	67.58^ab^	68.86^ab^	83.80^a^	3.28	78.16^b^	131.21^b^	152.71^b^	187.77^a^	14.02
T-AOC (μmol/g prot.)	5.54^b^	7.38^b^	7.47^b^	10.59^a^	0.52	4.86^b^	7.65^ab^	7.61^ab^	10.96^a^	0.67
T-SOD (U/g prot.)	18.45^b^	27.54^ab^	24^ab^	39.47^a^	1.52	19.88^b^	23.15^b^	23.79^b^	32.18^a^	1.36
CAT (U/g prot.)	51.55^b^	67.25^a^	65.57^ab^	73.17^a^	2.60	66.01^b^	61.49^b^	73.93^ab^	90.25^a^	3.55
Heart											
MDA (nmol/g prot.)	2.29^a^	0.85^b^	1.19^ab^	0.90^b^	0.20	2.10^a^	0.83^b^	1.27^ab^	0.79^b^	0.19
GSH-Px (U/g prot.)	59.80^b^	73.31^ab^	68.61^ab^	80.80^a^	2.92	106.20^b^	171.28^a^	105.81^b^	218.20^a^	13.60
T-AOC (μmol/g prot.)	6.31^b^	10.18^a^	7.78^b^	10.99^a^	0.53	5.31^b^	8.92^ab^	10.44^a^	12.02^a^	0.76
T-SOD (U/g prot.)	21.00^b^	26.46^b^	26.55^b^	37.06^a^	1.65	21.75^b^	26.46^b^	23.49^b^	36.54^a^	1.62
CAT (U/g prot.)	51.69^c^	66.37^ab^	65.79^b^	78.49^a^	2.77	60.94^b^	66.37^b^	59.73^b^	94.21^a^	4.33
Pectoral muscle											
MDA (nmol/g prot.)	2.43^a^	1.14^b^	1.33^ab^	0.83^b^	0.21	2.05^a^	1.26^b^	1.09^b^	0.62^b^	0.15
GSH-Px (U/g prot.)	58.87^b^	70.74^ab^	73.27^ab^	84.27^a^	2.85	93.31^b^	189.99^a^	193.47^a^	234.14^a^	18.15
T-AOC (μmol/g prot.)	4.82^b^	6.34^b^	5.70^b^	10.07^a^	0.58	6.62^c^	8.43^ac^	11.03^a^	11.37^a^	0.62
T-SOD (U/g prot.)	20.06^b^	23.89^b^	23.15^b^	29.44^a^	1.05	26.78^b^	31.71^b^	30.41^b^	53.25^b^	2.85
CAT (U/g prot.)	53.72^c^	65.01^bc^	71.50^b^	82.39^a^	2.94	45.82^b^	71.40^a^	68.77^a^	81.61^a^	3.88

^a,b,c^ Means in the same row within the same day without a common superscript letter differed statistically (*p* < 0.05). SEM: Standard Error Mean. Glutathione peroxidase (GSH-Px), total antioxidant capacity (T-AOC), catalase (CAT), and total superoxide dismutase (T-SOD), malondialdehyde (MDA).

**TABLE 7:** Dietary effects of Q, VE, and Q + VE on the antioxidant status of the 1-day-old chicks.

Parameters	Day 7					Day 14				
	C	Q	VE	Q + VE	SEM	C	Q	VE	Q + VE	SEM
Serum											
MDA (nmol/ml)	1.70^a^	1.06^ab^	0.88^ab^	0.59^b^	0.14	2.06^a^	0.95^b^	0.74^b^	0.76^b^	0.16
GSH-Px ((U/mL)	94.47^b^	169.04^ab^	146.30^b^	288.25^b^	22.45	107.04^c^	174.61^bc^	226.80^ab^	263.45^a^	17.61
T-AOC (μmol/ml)	6.23^b^	8.68^b^	9.44^b^	14.50^a^	0.94	7.04^b^	9.94^ab^	9.91^ab^	13.82^a^	0.82
T-SOD (U/mL)	22.13^c^	32.04^b^	32.25^b^	39.89^a^	1.77	17.82^b^	26.16^b^	34.09^ab^	50.19^a^	3.72
CAT (U/mL)	54.70^b^	76.95^b^	78.20^ab^	111.49^a^	6.37	63.37^c^	88.02^ab^	73.68^bc^	93.17^a^	3.47
Liver											
MDA (nmol/g prot.)	2.59^a^	0.91^b^	0.96^b^	0.69^c^	0.22	1.66^a^	0.99^a^	0.96^a^	0.63^b^	0.13
GSH-Px ((U/g prot.)	126.16^b^	131.21^b^	193.47^ab^	259.14^a^	18.64	91.23^b^	129.49^ab^	161.59^ab^	220.24^a^	16.34
T-AOC (μmol/g prot.)	6.20^b^	8.92^ab^	9.28^ab^	14.16^a^	0.95	7.94^b^	8.67^b^	8.43^b^	15.11^a^	0.90
T-SOD (U/g prot.)	26.21^b^	34.29^ab^	31.50^ab^	43.14a	2.03	21.25^b^	26.78^b^	23.77^b^	34.66^a^	1.48
CAT (U/g prot.)	54.24^c^	62.54b^c^	66.11^b^	88.23^a^	3.40	98.03^b^	105.93^b^	126.47^ab^	221.70^a^	16.58
Heart											
MDA (nmol/g prot.)	1.77^a^	0.97^ab^	0.89^ab^	0.66^b^	0.15	2.35^a^	0.83^b^	1.79^ab^	0.83^b^	0.22
GSH-Px ((U/g prot.)	116.04^b^	164.61^ab^	183.55^ab^	255.70^a^	18.36	74.67^b^	130.94^ab^	141.88^ab^	184.27^a^	13.60
T-AOC (μmol/g prot.)	7.90^b^	11.65^ab^	10.44^b^	16.01^a^	0.89	7.85^b^	11.12^ab^	10.20^ab^	13.92^a^	0.70
T-SOD (U/g prot.)	23.10^b^	26.49^b^	23.49^b^	36.28^a^	1.67	23.82^b^	25.66^b^	29.00^ab^	39.69^a^	1.97
CAT (U/g prot.)	62.30^b^	66.01^b^	77.20^ab^	93.46^a^	3.78	90.62^c^	107.97^bc^	115.48^b^	218.51^a^	13.14
Pectoral muscle										
MDA (nmol/g prot.)	1.76^a^	0.88^b^	0.84^b^	0.72^b^	0.14	2.00^a^	1.25^b^	0.98^b^	0.67^b^	0.15
GSH-Px ((U/g prot.)	111.88^b^	159.84^ab^	168.31^ab^	252.71^a^	19.49	119.02^b^	202.00^a^	207.45^a^	268.29^a^	16.17
T-AOC (μmol/g prot.)	6.88^b^	8.43^b^	9.44^ab^	13.25^a^	0.77	4.87^b^	8.37^ab^	10.87^a^	11.37^a^	0.84
T-SOD (U/g prot.)	21.22^b^	24.29^ab^	24.36^ab^	33.70^a^	1.62	23.24^b^	32.25^ab^	28.34^b^	38.37^a^	1.72
CAT (U/g prot.)	47.89^b^	71.40^a^	64.04^ab^	82.50^a^	3.86	38.32^b^	85.06^a^	66.21^a^	78.54^a^	5.56

^a,b,c^ Means in the same row within the same day without a common superscript letter differed statistically (*p* < 0.05). SEM: Standard Error Mean. Glutathione peroxidase (GSH-Px), total antioxidant capacity (T-AOC), catalase (CAT), and total superoxide dismutase (T-SOD), malondialdehyde (MDA).

**TABLE 8:** Dietary effects of Q, VE, and Q + VE on the antioxidant status of the 14-day-old chicks.

Parameters	Day 7					Day 14				
	C	Q	VE	Q + VE	SEM	C	Q	VE	Q + VE	SEM
Serum										
MDA (nmol/g ml)	1.69^a^	0.88^b^	0.79^b^	0.52^b^	0.13	2.13^a^	0.75^b^	0.82^b^	0.57^b^	0.69
GSH-Px (U/g mL)	78.67^b^	143.44^ab^	149.38^ab^	224.27^a^	17.18	98.56^b^	203.25^a^	173.31^a^	234.33^a^	16.06
T-AOC (μmol/g ml)	7.40^b^	11.87^b^	11.20^b^	18.95^a^	1.20	9.24^c^	12.98^b^	13.11^b^	19.07^a^	0.93
T-SOD (U/g mL)	22.52^b^	33.20^a^	33.05^a^	41.35^a^	2.01	23.40^b^	30.94^b^	29.36^b^	49.03^a^	2.65
CAT (U/g mL)	57.59^b^	93.91^a^	79.23^ab^	94.98^a^	4.80	43.38^c^	80.18^b^	74.46^b^	102.41^a^	5.92
Liver										
MDA (nmol/g prot.)	2.45^a^	1.38^b^	0.96^b^	0.59^b^	0.21	2.18^a^	0.61^b^	1.03^b^	0.58^b^	0.20
GSH-Px (U/g prot.)	117.71^b^	108.07^b^	204.32^a^	219.30^a^	13.34	94.84^b^	189.65^a^	152.71^ab^	213.45^a^	14.52
T-AOC (μmol/g prot.)	4.78^c^	7.80^bc^	10.09^b^	14.84^a^	1.07	9.10^c^	11.48^bc^	12.36^b^	17.62^a^	0.87
T-SOD (U/g prot.)	23.20^b^	30.42^ab^	31.26^ab^	37.19^a^	1.59	23.65^b^	29.44^ab^	29.09^ab^	38.53^a^	1.76
CAT (U/g prot.)	60.62^b^	83.77^a^	73.93^ab^	87.17^a^	3.32	50.35^b^	78.99^a^	88.93^a^	98.51^a^	5.18
Heart										
MDA (nmol/g prot.)	1.88^a^	0.93^b^	0.73^b^	0.45^b^	0.64	2.13^a^	1.03^b^	0.88^b^	0.84^b^	0.17
GSH-Px (U/g prot.)	102.52^b^	171.28^a^	105.81^b^	218.20^a^	14.04	96.11^b^	193.25^a^	140.81^ab^	201.83^a^	14.83
T-AOC (μmol/g prot.)	6.29^b^	6.85^b^	8.28^b^	14.43^a^	3.95	9.44^c^	11.36^b^	11.94^bc^	15.51^a^	0.28
T-SOD (U/g prot.)	25.59^b^	28.96^b^	29.97^b^	37.44^a^	1.29	22.88^b^	31.46^ab^	27.99^ab^	35.54^a^	1.61
CAT (U/g prot.)	58.87^b^	89.16^a^	77.23^ab^	97.48^a^	4.55	61.59^b^	81.25^ab^	69.37^ab^	98.21^b^	4.24
Pectoral muscle										
MDA (nmol/g prot.)	2.23^a^	1.31^b^	0.82^b^	0.72^b^	0.17	1.97^a^	0.77^b^	0.84^b^	0.51^b^	0.16
GSH-Px (U/g prot.)	116.52^b^	157.74^ab^	162.97^ab^	246.64^a^	16.66	110.88^b^	181.34^ab^	193.47^ab^	242.87^a^	15.85
T-AOC (μmol/g prot.)	8.69^b^	9.44^b^	9.51^b^	18.62^a^	1.13	9.34^b^	11.08^b^	11.44^b^	16.94^a^	0.82
T-SOD (U/g prot.)	22.55^c^	29.95^b^	29.80^b^	37.35^a^	1.47	25.37^b^	31.71^ab^	30.41^ab^	36.90^a^	1.49
CAT (U/g prot.)	55.88^b^	77.18^b^	72.21^b^	103.91^a^	5.28	62.71^b^	73.70^b^	75.70^b^	96.49^a^	3.69

^a,b,c^ Means in the same row within the same day without a common superscript letter differed statistically (*p* < 0.05). SEM: Standard Error Mean. Glutathione peroxidase (GSH-Px), total antioxidant capacity (T-AOC), catalase (CAT), and total superoxide dismutase (T-SOD), malondialdehyde (MDA).

### Effects of Dietary Q, VE, Q + VE on the mRNA Expression of Liver Antioxidant Genes of Embryo, Chicks at day-1 and day 14

The mRNA expression of the liver antioxidant genes of the embryo, chicks at day-1 and day-14 is represented in Table 9. The results showed that the combination of Q and VE increased the expressions of the antioxidant genes including deiodinase 1 (*DIO-1*), glutathione peroxidase 1 (*GPx1*), glutathione peroxidase 4 (*GPx-4*), superoxide dismutase 1 (*SOD-1*), and glutathione peroxidase 2 (*GPx-2*) in the liver of the embryo, day 1 and 14 old chicks compared to the control group at all-time points (*p* < 0.05).

**TABLE 9:** Effects of dietary Q, VE, Q + VE on mRNA expression of liver antioxidant genes of the embryo, chicks at day-1 and day 14.

Parameters	Day7					Day 14				
	C	Q	VE	Q + VE	SEM	C	Q	VE	Q + VE	SEM
Embryo’s liver										
GPx-1	1.05^c^	1.94^bc^	2.13^b^	3.28^a^	0.16	0.74^b^	2.96^a^	3.18^a^	3.76^a^	0.20
GPx-4	1.09^b^	2.93^a^	3.34^a^	3.56^a^	0.19	0.91^b^	2.70^a^	2.67^a^	3.33^a^	0.19
DIO-1	1.02^b^	1.62^b^	1.53^b^	2.97^a^	0.15	0.99^b^	2.30^a^	2.24^a^	2.79^a^	0.14
SOD-1	1.00^b^	3.04^a^	2.54^a^	3.36^a^	0.22	1.02^c^	3.18^a^	2.26^b^	3.23^a^	0.16
GPx-2	1.09^c^	2.57^b^	1.87^bc^	3.60^a^	0.16	0.98^b^	2.40^a^	2.44^a^	3.09^a^	0.17
1-Day old chick’s liver										
GPx-1	1.05^b^	2.31^a^	2.58^a^	3.00^a^	0.16	1.03^c^	3.60^ab^	3.08^b^	4.23^a^	0.18
GPx-4	1.01^b^	2.29^a^	2.58^a^	3.31^a^	0.17	1.05^b^	3.26^a^	3.05^a^	3.50^a^	0.18
DIO-1	1.06^b^	2.69^a^	2.49^ab^	3.38^a^	0.22	1.01^c^	3.03^b^	3.31^ab^	4.26^a^	0.20
SOD-1	1.00^b^	2.88^a^	3.46^a^	3.93^a^	0.21	1.03^c^	2.95^b^	3.24^ab^	3.84^a^	0.17
GPx-2	1.04^b^	2.69^a^	2.49^a^	3.38^a^	0.22	1.09^c^	2.02^b^	2.95^a^	3.05^a^	0.15
14-Day old chick’s liver										
GPx-1	1.06^b^	2.63^a^	2.74^a^	3.13^a^	0.14	1.00^c^	2.11^b^	2.26^ab^	3.32^a^	0.17
GPx-4	1.09^b^	3.01^a^	3.06^a^	3.32^a^	0.15	1.09^b^	1.87^ab^	1.62^b^	2.84^a^	0.16
DIO-1	1.08^c^	3.07^b^	2.99^b^	3.84^a^	0.13	1.03^c^	2.03^ab^	1.73^bc^	2.68^a^	0.14
SOD-1	1.06^c^	2.70^b^	2.25^bc^	4.03^a^	0.20	1.00^c^	2.32^b^	1.62^bc^	2.98^a^	0.16
GPx-1	1.02^b^	1.57^ab^	1.68^ab^	3.34^a^	0.14	1.05^b^	3.15^a^	3.08^a^	3.45^a^	0.19

^a,b,c^ Means in the same row within the same day without a common superscript letter differed statistically (*p* < 0.05). SEM: standard error mean.

## Discussion

Numerous studies have reported the association between storage time and changes in egg characteristics (Mohiti-Asli et al., 2008; Demirel and Kırıkçı, 2009; Günhan and Kirikçi, 2017). Prolong storage of eggs increases the egg weight loss, early embryonic mortality, decreased the fertility and hatchability of set/fertile eggs, chick weight, and ratio of healthy chicks (Yang et al., 2020). Egg weight loss (mostly water loss) is a key factor for incubation (Romao et al., 2008). This parameter is used in assessing the vital gas exchange (Rahn et al., 1979), and its association with embryonic development and metabolism (Rahn and Ar, 1980; Burton and Tullet, 1983). Breeder age has been linked with elevated egg weight loss during storage (Brake et al., 1997; Tona et al., 2001). For example, a study by Reis et al. (1997) reported that eggs obtained from aged hens (50-weeks breeder age) were likely to lose weight compared to eggs from younger hens, but less in percentage compared to eggs from younger birds (32-weeks breeder age). Many studies also found that as egg storage time increases the percentage of egg weight loss increases (Fasenko et al., 2001; Reijrink et al., 2009, 2008; Goliomytis et al., 2015).

The results in this study, showed that storage time (7 and 14 days) affected the egg characteristics such as egg weight, yolk weight, and yolk ratio, however, the feeding of maternal dietary Q + VE reduced the egg and yolk weight loss associated with old age and prolong storage. These results were consistent with the study by Yang et al. (2020), which reported that hen’s vitamin E improved egg yolk characteristics. Moreover, Amevor et al. (2021b), also reported that supplementation of dietary antioxidants improved egg characteristics and quality.

Embryo viability and the probability that a viable chicken will be hatched from an egg is linked with the developmental stage of the embryo at oviposition (Fasenko et al., 2001), which is in turn is decided by the egg clutch position and passage rate through the oviduct during egg formation. The egg clutch position and passage rate are influenced by the age of the breeder flock (Reijrink et al., 2008). Fasenko et al. (2001), which revealed that for an embryo to withstand the negative effect associated with prolong storage, the presence of an optimal morphological stage of the embryo is needed. Moreover, prolong egg storage decreases embryo viability. A study by Christensen et al. (2002) investigated the effect of storage (1 and 14 days) on two breeder flock ages (34 and 53 weeks) and found that at hatch, the quality of chicks from stored eggs laid by older breeder hens was highly decreased than that of the young breeders. Similarly, Tona et al. (2004) also reported that, 7 days of storage eggs resulted in a remarkable decrease in saleable chickens in older breeders (45 weeks; 14.3%) than in younger breeders (35 weeks; 0.81%). In addition, they observed that the number of malformed embryos noted with long-stored eggs (14 days) were higher with the older flock (49 weeks) compared to the younger flock (31 weeks). In the current study, we observed that age and storage time (7 and 14 days) affected the embryos viability, characterized by an increased embryonic mortality at both the mid and late stages of development, however, the feeding of maternal dietary Q + VE remarkably reduced embryonic mortality and increased the chick’s weight.

Hatchability and fertility rates are greatly dependent on the breeder flock age (O’Sullivan et al., 1991; Latour et al., 1996; Koppenol et al., 2015). Fertility, hatchability of set eggs (Zakaria et al., 2005; Almeida et al., 2008; Yilmaz and Boxkurt, 2009), and hatchability of fertile eggs in general declines as breeder hen ages (Elibol et al., 2002; Zakaria et al., 2005; Yilmaz and Bozkurrt, 2009). This was due to an increased in the percentage of late dead embryos (Elibol et al., 2002). This may be related to the fact that embryos derived from eggs laid by old breeder hens produce more heat from day 16 of incubation (due to the larger yolk size) compared to embryos from eggs laid by younger breeder hens (Nangsuay et al., 2013). Therefore, the need for the development of cellular and molecular mechanisms to reduce the rate of early embryonic mortality associated with egg storage has been raised by many researchers (Fasenko, 2007; Hamidu et al., 2011; Dymond et al., 2013). Our results showed that storage time (7 and 14 days) reduced fertility and hatchability of the eggs in the control group, however, the maternal feeding of Q + VE improved the fertility and hatchability at both time points. These results were consistent with studies by Urso et al. (2015), and Yang J. et al. (2018), which reported that the supplementation of vitamin E, selenium or quercetin improves fertility and hatchability in chickens, as well as other studies that reported that the interaction of dietary vitamins A and E exerted protective effects in layer hens during high ambient temperature by promoting blood hematological and biochemical indices (Abd El-Hack et al., 2017; Abd El-Hack et al., 2019).

Oxidative stress is associated with poor embryo growth, hatchability, and chick development (Araújo et al., 2019; Shafienejad et al., 2021). Numerous studies have reported that prolong storage of eggs increase oxidative stress of yolk and embryos (Bakst et al., 2016; Nasri et al., 2020). From this present study, we observed that prolong storage (7 and 14 days) increased the levels of oxidative stress parameter MDA and reduced the levels of antioxidants indicators GSH-Px, T-AOC, T-SOD, and CAT in the egg yolk of the control group. However, the supplementation of dietary Q + VE into the maternal diet decreased the levels of the egg yolk MDA, whereas the GSH-Px, T-AOC, T-SOD, and CAT levels in the egg yolk were increased at both day 7 and 14. Similar results were obtained by Khaligh et al. (2018), who reported that the supplementation of dietary quercetin, chrysin, and ascorbic acid improved yolk antioxidant status. Another study showed that dietary supplemetation of Echinacea purpurea (EP) meal promotes productive and reproductive performance of breeder ducks during summer season through alleviating oxidative stress (Awad et al., 2021). In addition, 7 and 14 days of storage increased the oxidative stress of the embryo by increasing the production of ROS, which reflected in the higher levels of MDA and lowers the levels of GSH-Px, T-AOC, T-SOD, and CAT in the liver, heart, and pectoral muscle, as well as downregulates the antioxidant genes including *GPx-1*, *GPx-2*, *GPx-4*, *DIO-1*, and *SOD-1* in the liver of the embryos in the control group, but the hen’s dietary Q + VE reduced the MDA and increased the levels of antioxidant parameters in the embryo’s liver, heart, and pectoral muscle, and upregulated antioxidant related genes *GPx-1*, *GPx-2*, *GPx-4*, *DIO-1*, and *SOD-1* in the liver of the embryo. This was consistence with the studies by Tang et al. (2006) and Surai and Sparks (2001), which reported that the combination of daidzein and quercetin, and vitamin E and carotenoids, respectively protects chicken’s embryo from oxidative stress. Moreover, studies have shown that deleterious effects of pollutants such as cadmium (Cd) in grower Japanese quail could be reversed by the supplementation of vitamin E which promotes liver function, growth performance, and carcass characteristics (Abou-Kassem et al., 2016).

Furthermore, we observed that maternal dietary Q + VE improved the antioxidant status of day 1 and 14 chicks at both time points. The MDA levels in the blood, liver, heart, and pectoral muscles were remarkably elevated, whereas the levels of antioxidants indices GSH-Px, T-AOC, T-SOD, and CAT as well as mRNA expression of antioxidant genes *GPx-1*, *GPx-2*, *GPx-4*, *DIO-1*, and *SOD-1* in the liver of chicks (day 1 and 14) were reduced in the control group, however, the supplementation of the combination of dietary Q + VE reverted the oxidative stress effects in the blood, liver, heart, and pectoral muscles by elevating the levels of antioxidants indices GSH-Px, T-AOC, T-SOD, and CAT, and upregulates the antioxidant genes *GPx-1*, *GPx-2*, *GPx-4*, *DIO-1*, and *SOD-1* at both time points. These results were consistent with the studies by Ulaiwi (2018) and Torki et al. (2018) which reported that the combination of levamisole, vitamin E, and selenium and quercetin, oat hulls, β-glucans, lysozyme, and fish oil, respectively reduce oxidative stress in broiler chicks.

In conclusion, the supplementation of maternal diet with Q + VE increased hatchability by decreasing early embryonic mortality, which was achieved by increasing the antioxidant status of the egg yolk, liver, heart, and pectoral muscle of the embryo, as well as increased the chick’s quality by improving the antioxidant status of the blood, liver, heart, and pectoral muscle of 1-day-old and 14-day-old chicks. These findings suggested that the supplementation of the combination of Q and VE into the aged hen’s diet exert beneficial synergistic effects on egg yolk, embryo, and chicks produced from prolong storage eggs, therefore, Q + VE could be used as a dietary measure to enhance hatchability and chick quality in poultry production.

## Data Availability

The original contributions presented in the study are included in the article/Supplementary Material, further inquiries can be directed to the corresponding author.
